# Indoor UWB Positioning and Position Tracking Data Set

**DOI:** 10.1038/s41597-023-02639-5

**Published:** 2023-10-26

**Authors:** Klemen Bregar

**Affiliations:** Institut Jožef Stefan, Department of Communication Systems, Ljubljana, 1000 Slovenia

**Keywords:** Electrical and electronic engineering, Information technology, Computer science

## Abstract

Indoor positioning has become a hot topic in various fields, such as industry, healthcare, and commerce. Ultra-wideband (UWB) radio technology provides a cost-effective solution for range-based positioning, offering exceptionally high accuracy and precision. Its ultra-high temporal resolution enables range measurements with accuracy of a few centimeters. To develop and evaluate range-based positioning systems, we collected measurements in four different indoor environments using eight fixed devices and one mobile positioning device. To eliminate the fluctuation of walking speed from the data, we pre-defined a path in each indoor environment, similar to the human walking path, which was sampled at equidistant positions. We collected multiple range measurements and channel impulse response (CIR) data at each tag position on the path. The resulting dataset supports the development of range-based positioning and position tracking algorithms with various combinations of network topologies and anchor-tag combinations. We have also provided a full set of data analysis tools that enable the reproducibility of results and serve as a basis for further development of range-based UWB positioning algorithms.

## Background & Summary

Indoor positioning, also known as indoor localization, is the process of determining the position of a person or object within a building or enclosed space using various technologies such as wireless communication technologies, acoustic, infrared and techniques based on computer vision^1^. Several technologies and approaches exist that support indoor positioning and several solutions emerged during past two to three decades that implement indoor positioning^2^. Indoor positioning systems typically use a combination of wireless technologies such as Wi-Fi, Bluetooth, ultra-wideband (UWB), radio-frequency identification (RFID), different sensors such as accelerometers and gyroscopes and various positioning algorithms that combine them into solutions that estimate the position with high accuracy. The performance of Global navigation satellite systems (GNSS) for positioning in indoor environments is generally limited by the effects of non-line-of-sight (nLoS) signal propagation and therefore high degree of signal attenuation and by the effects of multipath propagation^1^.

Indoor positioning has two major applications in the context of the internet of things (IoT) networks and devices. It can be found as a standalone IoT service or it can serve as a supporting service in a more general-purpose IoT deployment. It is present in the industrial environments for tracking personnel and equipment,for tracking and navigation of automated guided vehicles (AGV) and for increasing and ensuring the workplace safety (e.g. geofencing, access management etc.)^2^. Indoor positioning systems (IPS) can also play important part in medical environment by enabling tracking the personnel and equipment. It received increased attention during the COVID-19 pandemic as an enabling solution for several social distancing and exposure tracking concept implementations^3^. One of the most prominent customer assistance services is indoor navigation which can help customers in shopping malls, libraries and airports find the way to desired product sections, to the book they are seeking or to the right terminal in the airport^4^.

Amongst the technologies supporting indoor positioning and navigation, radio frequency (RF) based approaches found a particularly significant position due to their ubiquity and low-cost compared to complex computer vision and optical positioning systems. First group of RF positioning systems use geometric properties of RF links between devices to estimate the position, where there are distance-based techniques (lateration), such as time-of-arrival (ToA), time-difference-of-arrival (TDoA), RSS-based range estimation and two-way ranging (TWR) and angle-based techniques (angulation), such as angle-of-arrival (AoA) technique^5^. The second group of RF positioning techniques is based on a scene analysis approaches, which are also known as RF fingerprinting positioning approaches^5^.

Based on the availability and their ubiquity (relatively inexpensive and simple to implement in hardware) the most easily employed techniques for indoor positioning are techniques based on the received signal strength (RSS)^6^. Technologies like Wi-Fi and Bluetooth are present in most of smart devices like smart phones and are therefore available for development of indoor positioning and navigation systems for consumers without heavy investments in the infrastructure. One of the simplest and most widely used approaches for RSS-based indoor positioning is the approach where distance between the transmitter and the receiver is estimated based on a simple logarithmic path loss model^2^. The logarithmic path loss model is presented by the Eq. (1),

1 $$PL(d)=P{L}_{0}+10n\;{\log }_{10}\left(\frac{d}{{d}_{0}}\right)$$

where *PL*(*d*) is a path loss at distance *d*, *PL*_0_ is a path loss value at reference distance *d*_0_ (typically *d*_0_=1 m), *n* is the path loss exponent and *d* is distance between the transmitter and the receiver. Path loss exponent can be selected based on the type of the environment (can be found in literature^7^) and typically varies between *n*=2 for free space propagation and *n*=4 in indoor environments^2^. To work directly with the RSS values, path loss model can be transformed into a form, where RSS power is being estimated using path loss exponent *n*, which is presented by the Eq. (2)^8^,

2 $$RSS(d)=-10n\;{\log }_{10}(d)+C$$

where *RSS*(*d*) is received power in dBm at distance *d*, *n* is path loss coefficient, *d* is the distance between the transmitter and the receiver and *C* is a fixed constant (intercept) of a linear function that we fit to graph of *RSS* measurements at different distances *d*. The method is simplistic but lacks accuracy because the communication channel is nonstationary in time. The statistics of channel change even in case when transmitter and receiver are stationary because of the constant changes in the environment (e.g. movement of people, high diversity of propagation paths with changes in positions)^9^. Therefore, high positioning accuracy is achievable only with the use of complex algorithms which often require extensive site surveys for calibration^2^.

Unlike range estimation techniques based on RSS, time-based ranging techniques offer higher ranging accuracy. The accuracy of a time-based approach can be improved by increasing the signal-to-noise ratio (SNR) and by increasing signal bandwidth^10^. The interactions between devices in UWB domain are performed by sending or exchanging the information in a form of trains of very short pulses (<1 ns) which defines a signal bandwidth that is greater than 500 MHz^2^. That gives the UWB systems very high temporal resolution which gives UWB systems ability to separate direct path signals from the delayed multipath components. Analysis on a lower bound of ToA variance in a multipath-free environment shows that doubling either the bandwidth or doubling the transmit power will cut the ToA variance in half^6^. The high temporal resolution makes UWB systems able to measure time-of-flight (ToF) very accurately and therefore makes UWB systems perfect for ToF-based positioning techniques. While the achieveable ToF measurement accuracy is very high under ideal conditions, the level of clock synchronization between the nodes becomes an important factor in ToF measurement accuracy^10^.

Three methods are most commonly found in a group of time-based ranging techniques: ToA, TDoA and TWR. ToA techniques are based on the nodes with internal clocks fully synchronised to the master network clock. To measure the ToF, transmitter attaches a transmit timestamp to the packet and sends it to the receiver, where receiver timestamps the received packet with a receive timestamp. Because the internal clocks of all devices in the network are synchronised to one master clock, ToF is calculated by subtracting the transmit timestamp from the receive timestamp. However, the main source of ranging error comes from the node clock synchronisation mismatch, where each nanosecond error in ToF means roughly 30 cm of ranging error^6^. Precise clock synchronisation is also costly in communication overhead needed for clock synchronisation wich introduces additional energy consumption.

When the time synchronisation is available only between reference nodes (anchors) and not mobile nodes (tags), a TDoA techniques can be deployed^10^. In this case, a packet (beacon) is transmitted by the mobile node (tag) and received by all reference nodes in communication range. Each anchor marks the received message with the global timestamp (anchors are synchronised in TDoA scheme) and reports the receive timestamp to the positioning engine (central location in the network). All anchors that received the message report the receive timestamps to the positioning engine. From the differences between the timestamps, the node’s position can be estimated. For each TDoA measurement, the mobile node lies on a hyperboloid with a constant range difference between the two reference nodes (anchors)^5^. By limiting the synchronisation requirements only to the anchors, the amount of communication for the mobile node (tag) is reduced which simplifies the node’s firmware complexity and reduces the power consumption.

In the positioning systems without any time synchronisation between the nodes, TWR ranging schemes exist that prowide range measurements by consecutivelly timestamping message during the round-trip propagation^10^. The first node transmits a message which already contains the local transmit timestamp. The message is received by the second node and the message is being timestamped by the local receive timestamp. The second node responds with the same message back to the first node and attaches response timestamp. The response message is being received at the first node and timestamped by the receive timestamp. By having all four timestamps, ToF can be calculated from them without having any synchronisation between the nodes. Local clock deviations are being eliminated by having four timestamps from the TWR measurement which enables ToF measurements without clock synchronisation. Each ToF measurement requires more messages to be exchanged during each measurement cycle compared to synchronised approaches (e.g. TDoA), but the simplicity of the implementation and absent requirement for synchronisation makes it feasible to be used in low-power sensor networks by using simpler devices.

To close the vertical from ranging to positioning, there is one possible algorithm that can be used in range-based positioning systems. The algorithm is called multilateration or simply lateration. It can be defined by mathematically describing the individual ranges between tag and individual anchor as Euclidean distance (Eq. (3))

3 $${r}_{i}=\sqrt{{({x}_{i}-x)}^{2}+{({y}_{i}-y)}^{2}},$$

where *r*_i_ is the measured range between the i-th anchor (*x*_*i*_*, y*_*i*_) and the tag (*x*, *y*) with an unknown position.

By writing the equations for all tag-anchor pairs we get a predefined system of equations. To linearize the resulting system of non-linear equations, we introduce a new variable as in Eq. (4).

4 $$R={x}^{2}+{y}^{2}$$

The position can be now estimated by the Eq. (5).

5 $$\widehat{{\boldsymbol{\theta }}}={\left[\begin{array}{ccc}x & y & R\end{array}\right]}^{T}$$

The resulting predefined system of linearized equations is presented in a matrix form as **H** (Eq. (6)) and as **x** (Eq. (7)).

6 $${\bf{H}}=\left[\begin{array}{ccc}-2{x}_{1} & -2{y}_{1} & 1\\ -2{x}_{2} & -2{y}_{2} & 1\\ \vdots  & \vdots  & \vdots \\ -2{x}_{N} & -2{y}_{N} & 1\end{array}\right]$$

7 $${\bf{x}}=\left[\begin{array}{ccc}{\widehat{d}}_{1}^{2} & -{x}_{1}^{2} & -{y}_{1}^{2}\\ {\widehat{d}}_{2}^{2} & -{x}_{2}^{2} & -{y}_{2}^{2}\\  & \vdots  & \\ {\widehat{d}}_{N}^{2} & -{x}_{N}^{2} & -{y}_{N}^{2}\end{array}\right]$$

The resulting predefined system of linear equations can be used for position estimation by the use of least squares estimator in a normal form as is presented by the Eq. (8).

8 $$\widehat{{\boldsymbol{\theta }}}={({{\bf{H}}}^{T}{\bf{H}})}^{-1}{{\bf{H}}}^{T}{\bf{x}}$$

The aim of this paper is to give the scientific and engineering community a well-described data set that will enable developing the algorithms for nLoS-induced ranging error mitigation to improve the accuracy of range-based indoor positioning systems. The dataset was created to analyze several range-based positioning algorithms that can be used for precise indoor positioning services. The measurements were made in four different indoor environments where in each environment a trace of equally-distanced positions that resemble a human walking path were placed. Each position in a data set can be used for evaluation of stationary positioning algorithms and also for dynamical position tracking algorithms. For each position on a walking path several channel sounding measurements in a form of channel impulse response (CIR) and measured range is collected for each of eight TAG-ANCHOR pairs. Depending on the actual position on a walking path, radio links of individual TAG-ANCHOR pairs are either in a line-of-sight (LoS) or in a nLoS situation. The contributions of each measurement to the final position accuracy is therefore dependent on conditions of all eight TAG-ANCHOR radio links. The collected ground truth positions and measured distances between TAG-ANCHOR pairs enabled us to develop positioning and tracking algorithms based on machine learning (ML) models for positioning accuracy improvements by ranging error mitigation.

We systematically layed down the Methods Section from introducing the measurement equipment, measurement network, to describing the UWB ranging procedure and describing the four indoor environment that were used in the process of the data set generation. We described the parameters that were used for the data collection and described the reasons for the limited selection of parameters covered in the data set and finalized the Section Methods by describing the data cleaning and data preprocessing procedures used to produce the published data set. In the Data Records Section we provide the description of data format, how the data is organized in the actual .*json* files and how the data set folder is organized including the description of floorplans and included technical validation programming scripts and sources that were used to prepare the figures and results for the paper. Technical Validation Section presents few angles on the data set that graphically present the quality of the data. The Section Usage Notes concludes the presented material by giving few practical examples for the usage of the data set that can be reproduced in full by running the scripts included in the technical validation subfolder of the data set.

## Methods

In this section the complete procedure to collect data is described including descriptions of measurement equipment used. The data set contains data from four indoor environments with ranging and CIR measurements for indoor positioning, tracking and ranging error estimation research.

### Measurement system

For the measurements in the UWB domain, we chose the low-cost, commercially available DecaWave DW1000^11^ (now Qorvo DW1000) UWB transceiver in the form of a certified and integrated RF module DWM1000^12^. The module has integrated ceramic antenna from Partron ACS5200HFAUWB^13^. The specifications of selected antenna and radiation patterns are available in a document^13^. We designed UWB measurement and communication device SNPN-UWB^14^ connecting the selected DWM1000 UWB module to the application microcontroller (MCU) STM32F103RE^15^. The application MCU controls the DWM1000 UWB module and implements a measurement communication protocol. Application MCU acts as a communication bridge between an embedded measurement computer that controls the UWB node operation and collects measurements using UART or USB communication interface.

The measurement nodes used to collect the measurements for this data set are based on a Raspberry PI 3 Model B + ^16^ embedded single board computer (SBC). Each measurement node consists of a SNPN-UWB device and a Raspberry PI 3 Model B + SBC with Wi-Fi connectivity enabled.

The measurement network used to create this data set consists of 9 measurement nodes. Measurement nodes are connected to a Wi-Fi access point. To control the measurements, a personal computer (PC) is connected to the same Wi-Fi network and runs a UWB measurement application. The MQ Telemetry Transport (MQTT)^17^ broker is installed on PC as a communication interface between the measurement application and the measurement nodes on the application level.

**Fig. 1:**
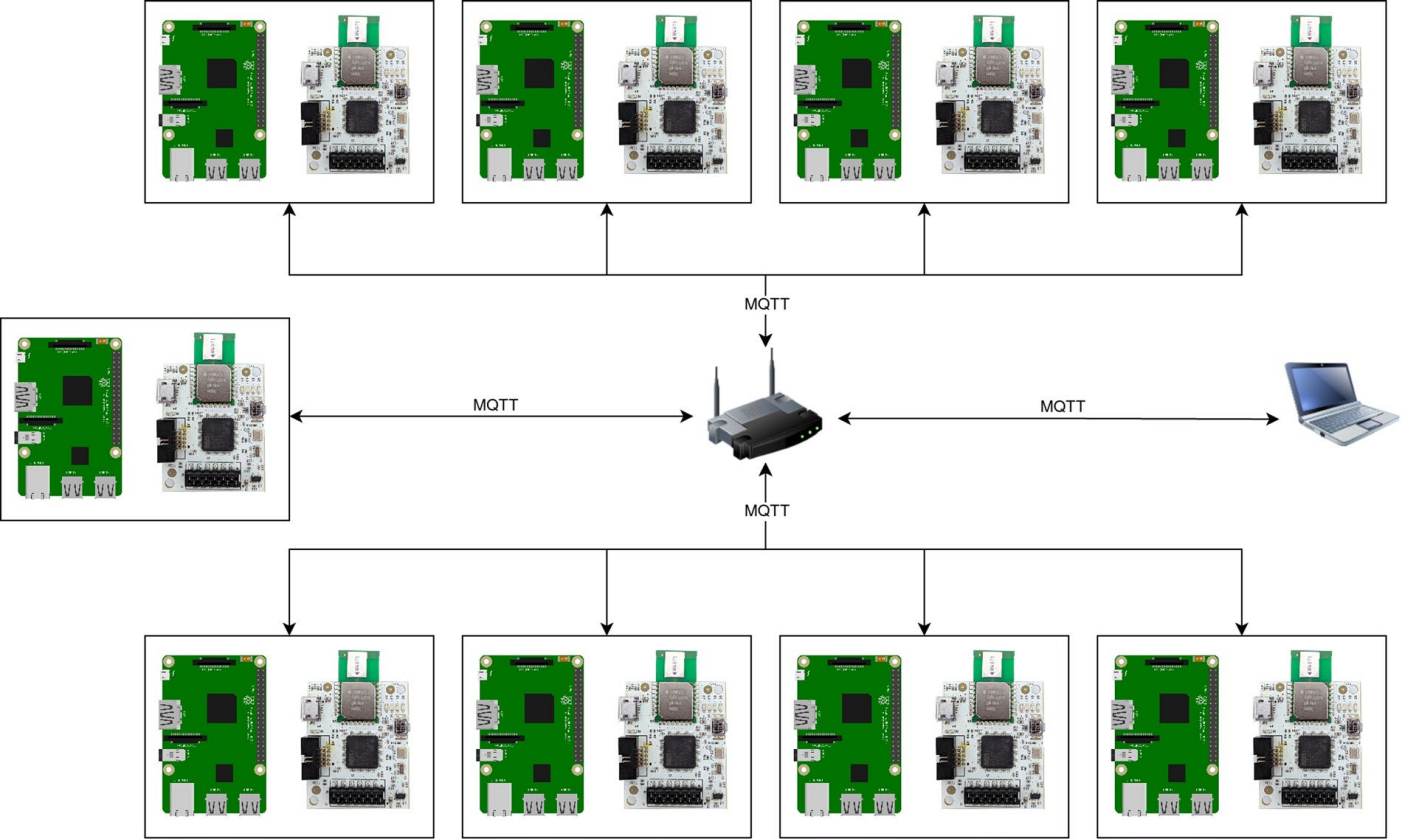
Measurement system architecture.

The UWB measurement application on PC connects locally to the MQTT broker and subscribes to the UWB topic. The measurement application on the individual measurement nodes connects to the MQTT^17^ broker on a PC via the Wi-Fi network and waits for the commands from the UWB measurements application. Figure 1 shows the complete measurement network including the measurement PC and the Wi-Fi access point.

### UWB Ranging

The DW1000 UWB transceiver provides physical access to the UWB communication medium. For the measurements described in this paper, we implemented a simple UWB communication protocol that implements asymmetric double-sided two-way ranging (ADS-TWR)^18^. In our implementation of the ADS-TWR measurement scheme, there are always two devices: the anchor device and the tag device. The anchor device is a UWB device with a static (fixed) position and thus represents the localization infrastructure. The tag device is a UWB device with a dynamically changing position and thus represents a device with an unknown position (we measure ToF to estimate the position of the tag device). The tag device initiates the ToF measurement because it wants to know the distance to the reference anchor device. The list of distances to several reference devices (anchors) can be used to estimate the position of the tag device. The UWB device pair that performs the ADS-TWR measurement process in our measurement network exchanges four UWB packets, the first three of which are used for the actual ToF measurement and the fourth for reporting the ToF measurement (range) to the tag device that initiated the ToF measurement process in the first place.

**Fig. 2:**
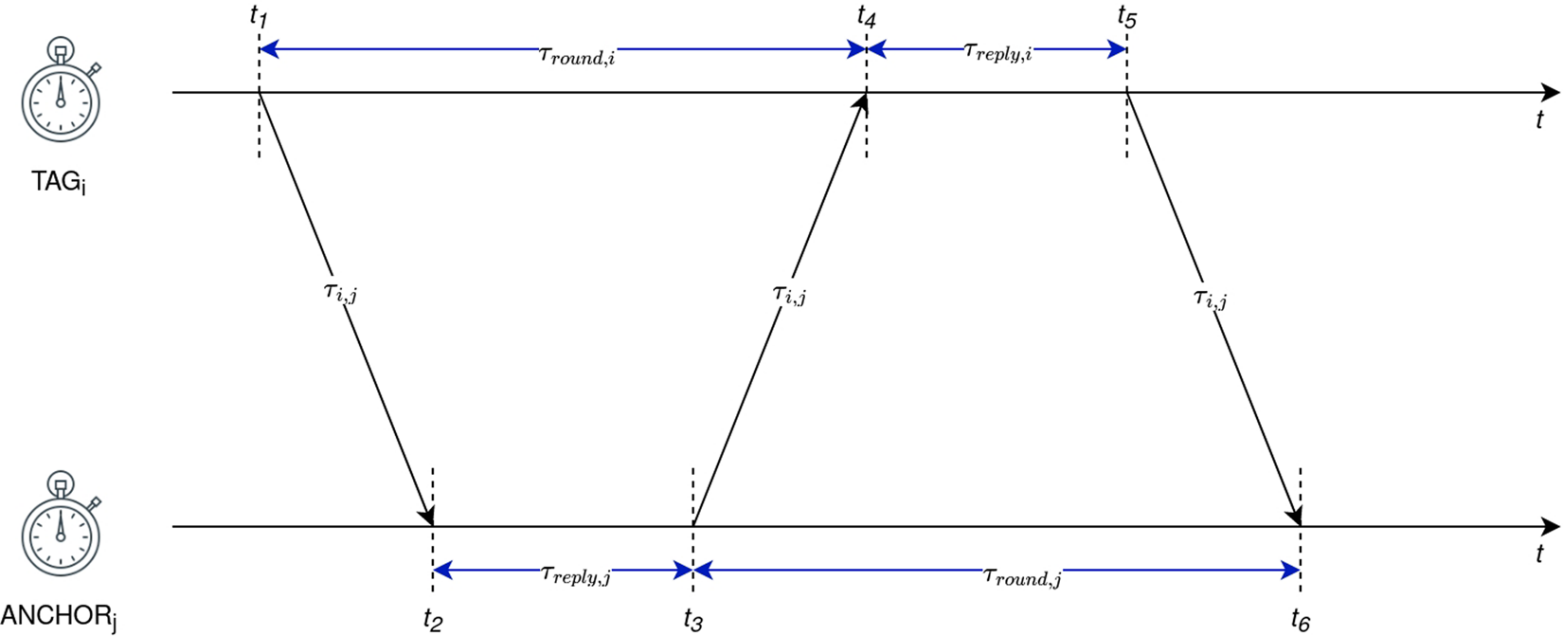
ADS-TWR timing presentation.

The ADS-TWR ranging technique used for the data set collection exchanges three messages to measure the ToF, where ADS-TWR scheme compared to the basic double-sided scheme reduces the error due to clock and frequency drift^18–20^. The process of measuring ToF with ADS-TWR is graphically represented in Fig. 2, where ADS-TWR is presented without antenna delays. *TAG*_*i*_ sends a message to *ANCHOR*_*j*_ at *t*_1_ which receives the message at *t*_2_. *ANCHOR*_*j*_ replies with the response message at *t*_3_ after waiting for the predefined response time *τ*_*reply,j*_. *TAG*_*i*_ receives the response message at *t*_4_ and sends a new response message at *t*_5_ after waiting for the predefined time *τ*_*reply,i*_, which is received by *ANCHOR*_*j*_ at ${t}_{6}$. Equation (9) presents a way, how a ToF (${\tau }_{i,j}$) can be calculated from the round trip times (RTT) and reply times *τ*_*round i*_, *τ*_*round j*_, ${\tau }_{reply,i}$ and ${\tau }_{reply,j}$. *τ*_*round,i*_ is a RTT for the first round trip starting at the *TAG*_*i*_ which can be calculated as ${\tau }_{round,i}={t}_{4}-{t}_{1}=2{\tau }_{i,j}+{{\rm{\tau }}}_{reply,j}$ (variable value depending on the propagation time) and ${\tau }_{reply,i}$ is the reply time of $TA{G}_{i}$ (fixed value). The values on the side of *ANCHOR*_*j*_ are defined very similarly, where ${\tau }_{round,j}={t}_{6}-{t}_{3}=2{\tau }_{i,j}+{\tau }_{reply,i}$ is a RTT at *ANCHOR*_*j*_ and ${\tau }_{reply,j}$ is the reply time of *ANCHOR*_*j*_.

9 $${\tau }_{i,j}=\frac{{\tau }_{roundi}{\tau }_{round,j}-{\tau }_{reply,i}{\tau }_{reply,j}}{{\tau }_{reply,i}+{\tau }_{reply,j}+{\tau }_{round,i}+{\tau }_{round,j}}$$

### Measurement environments

Four different indoor environments were selected for the measurement campaign to cover variable influence of indoor environments to the RF signal propagation. The selected environments have two representatives from the living (residential) environment, one from office environment and one from industrial environment full of RF signal reflecting surfaces and high amount of nLoS situations.

**Fig. 3:**
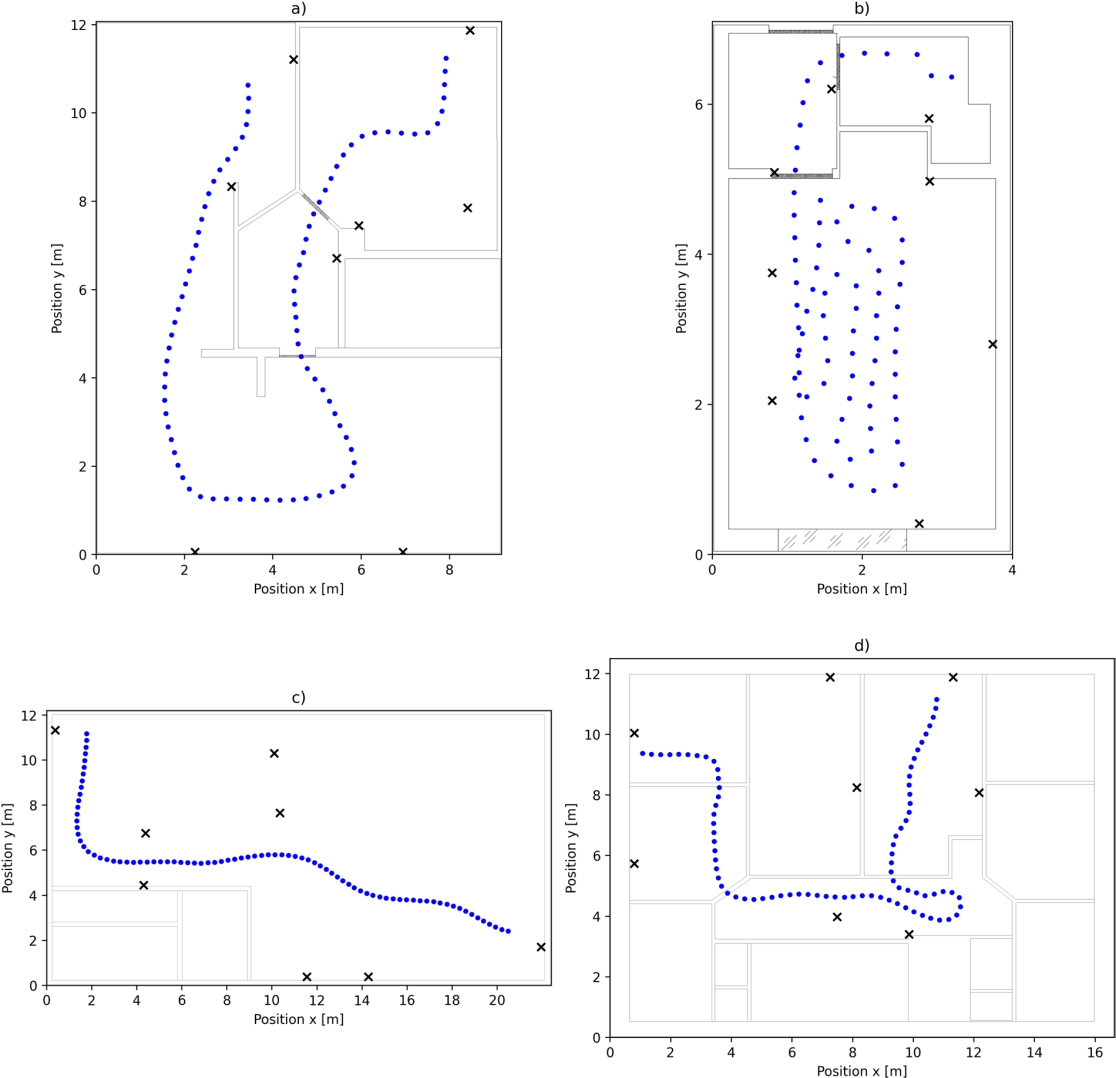
Floorplan of *environment0* (**a**), *environment1* (**b**), *environment2* (**c**) and *environment3* (**d**) with anchor positions and simulated walking paths. Positions on a simulated walking path are presented with blue dots while anchor positions are presented with black crosses.

First indoor environment included in the data set^21^ is designated as *environment0* and represents apartment in a residential house. The apartment occupies the entire floor in the house. The width of the floor plan is *w* = 9.18 and length is *l* = 12.06. Outside walls are made of brick and all inside walls except two on the right of the floor plan are made of a plaster and insulation material. The floorplan of the *environment0* is presented in the Fig. 3a.

Second indoor environment in a data set called *environment1* represents small apartment in a bigger apartment building with dimensions *w* = 3.60 and *l* = 6.69. All outside walls are made of concrete while inside walls are made of plaster and insulation material. The floorplan of *environment1* is presented in Fig. 3b.

*Environment2* represents bigger workshop full of machines and equipment. The outside walls of a workshop are made of brick and partially glass and most of the equipment is made of metal and thus highly reflective for the radio waves. The width of the workshop is *w* = 21.96 *m* and length is *l* = 11.85 *m*. The floorplan of *environment2* is presented in Fig. 3c.

The fourth indoor environment called *environment3* represents an office environment which takes complete floor in an office building. Width of office environment is *w* = 15.37 and length is *l* = 11.50. Outside walls of the office environment are made of concrete where inside walls are made of plaster and insulation material. The floorplan of *environment3* is presented in Fig. 3d.

### Measurement procedure

In each indoor environment we positioned eight anchor nodes at reference positions that are not colinear and are spread enough through the environment to cover complete measurement area. Most of anchor positions are located on the walls (environment borders). During the individual measurement campaign in a selected indoor environment tag node was consecutively placed at all predefined positions on a pre-defined walking path. The walking path is evenly sampled with equidistant points. This approach resembles constant walking speed and provides measurement (sampling) positions that are equally spaced spatially and temporarily.

Equal distances between positions on the walking path provide a base for a data set that can be used for evaluation of position tracking algorithms. All recorded UWB samples for each point on a walking path represent measurement to the same point without any artifacts induced by the motion of the tag device. The tag device, all anchor devices and the environment are static while taking measurements at the selected walking path position. Infinite number of measurements can be taken at each position without changing the channel measurement sampling rate. The only limit is the time we want to spend taking measurements at each position and amount of storage space we are allowed to consume.

To precisely position the tag device on a pre-defined points on a walking path we used laser-based distance measurement approach. Laser-based distance measurement devices provide precise and relative low-cost solution for distance measurement where consumer and professional devices easily approach accuracy in millimeter range. We used a laser-based distance measurement device Bosch GLM 120 C^22^ with claimed ±1.5 mm accuracy. Reference position of a tag on a walking path was set by measuring distance to reference points in the particular indoor environment and moving the tag until the pre-defined reference position on a walking path was reached.

The general measurement procedure was repeated with tag placed at all positions on a walking path. For each tag position measurement operator entered a reference tag position in the selected indoor environment coordinate system and started the measurement application. Measurement application looped through the list of 8 anchors and 6 available UWB communication channels and collected 31 measurements for each tag-anchor-channel combination. Therefore, for each position at simulated walking path, we recorded 48 files with 31 measurements in each, which gives us 1488 measurements per one position on a simulated walking path. Table 1 presents indoor environments with corresponding number of points on a simulated walking path and total count of measurements made in a particular environment before data preprocessing.

**Table 1:** Table of indoor environments, number of points on simulated walking path and number of measurements made in the particular environment.

Environment	Positions	Number of Measurements
environment0	85	126480
environment1	80	119040
environment2	84	124992
environment3	81	120528

### UWB radio settings

The DW1000 UWB radio support many communication configurations. The radio supports the configurations defined by the IEEE 802.15.4 standard and further extends the available configurations by adding proprietary options to expand the flexibility of the transceiver.

The pool of all transceiver setting combinations is too wast to be covered entirely in the data set. We selected configurations with pulse repetition frequency (PRF) of 64 MHz, one preamble code, preamble length of 4096 symbols, preamble acquisition chunk (PAC) of 64 symbols and data rate of 110 kbit/s. The PAC parameter defines the size of a group of preamble symbols which are correlated together in the preamble detection process while receiving an UWB frame. Preamble codes are defined in IEEE 802.15.4-2011 standard^23^ and referenced in DW1000 User Manual^24^.

Non-standard DecaWave proprietary start of frame delimiter (NSSFD) start of frame delimiter (SFD) sequence with the length of 64 symbols was used during the measurements for the data set. DW1000 transceiver supports the use of different SFD sequences than defined or suggested by the IEEE 802.15.4 standard to potentially optimize the performance if needed where standard compliance is not mandatory/required. The SFD pattern consists of a sequence of preamble symbols either not sent, sent in normal orientation or sent inverted. As it is stated in the DW1000 manual^24^, defining the reliable SFD sequence is a complex task and is not recommended for others than field experts. In a case where better performance is desired and standard compliance is not required, the manufacturer suggests the use of a Decawave proprietary non-standard SFD sequence.

The transmit power was set to the highest setting available at the DWM1000 transceiver. The available transmit power settings at DWM1000 are not defined by the absolute transmit power levels in but are defined by the transmit gain parameter. The transmit gain control range is 33.5 dB with 32 fine 0.5 dB control steps and 7 coarse 3 dB control steps. The transmit (TX) power for the collection of data in this data set was was set to 33.5 dB to extend the communication and measurement range to the highest possible level.

In real deployments the TX power is limited by the spectral emission regulations at −41.3 dBm/MHz which will limit the communication range. Transmit power can be increased when sending short packets at 6.8 Mbit/s, where single frame with a duration shorter than 1 ms is sent inside the time frame of 1 ms. Spectral emission regulation limit the spectral emissions per 1 MHz inside the time window of 1 ms at −41.3 dBm/Mhz. If the frame duration is shorter than 1 ms and only one frame is being sent inside the duration of 1 ms, the transmit power can be increased while maintaining the compliance to the spectral emission regulations.

Selected UWB radio combinations that were used to create the data set are collected in the Table 2. All parameters were already breafly described but for more extensive explanations, the device’s manual^24^ and the IEEE 802.15.4 standard^23^ documentation are the preffered sources of information.

**Table 2:** Table presents all radio settings that were used for measurements. Please note, that channel bandwidth information noted in the table are specified in the IEEE 802.15.4 standard but are limited by the physical limitations of the DW1000 radio. The DW1000 bandwidth is limited to 900 MHz.

CH	f [MHz]	BW [MHz]	PRF [MHz]	Preamble code	Preamble length	PAC	DR [kbit/s]
1	3494.4	499.2	64	9	4096	64	110
2	3993.6	499.2	64	9	4096	64	110
3	4492.8	499.2	64	9	4096	64	110
4	3993.6	1331.2*	64	17	4096	64	110
5	6489.6	499.2	64	9	4096	64	110
7	6489.6	1081.6*	64	17	4096	64	110

### Cleaning and preprocessing data

First thing after collecting the measurement data, we performed the initial data quality estimations and performed needed data cleaning and data preprocessing operations.

#### Remove outliers

Real data is always affected by containing some invalid data that is out of all reasonable ranges. That can happen by any reason but in our case, in some rare occasions the random individual measurements were off for a significant amount from the average range of the current mesurement cluster.

The data set was cleaned for outliers with a density-based spatial clustering of applications with noise (DBSCAN) clustering algorithm^25,26^ with parameters *ε* = 2.0 and *min_samples* = 5. Parameter *ε* defines the maximum distance between two samples for one to be considered in the neighbourhood of the another sample. Parameter *min*_*samples* defines a minimum number of samples in the neighbourhood of a point to be considered a core point of a particular cluster.

The outliers were removed from the data by going through all measurement files for each tag position, communication channel and anchor combination. Measured ranges and actual ranges from each measurement file were loaded into a 2D array and processed by a selected clustering algorithm. The samples not being a part of any cluster produced by the clustering algorithm were considered outliers and thus removed from the data set. The process of removing the outliers removed 5 outliers in *environment0*, 0 outliers in *environment1*, 2 outliers in *environment2* and 22 outliers in *environment3*.

#### Range offset compensation

DW1000 UWB transceiver allows very accurate timestamping of packets leaving and arriving at the transceiver. These timestamps, together with Time of Flight (ToF), include also unspecified delays that occur during the time of received signal propagation from antenna to the internal circuitry and those that occure while propagating from internal circuitry to the antenna. These delays are designated as receive and transmit antenna delays.

**Fig. 4:**
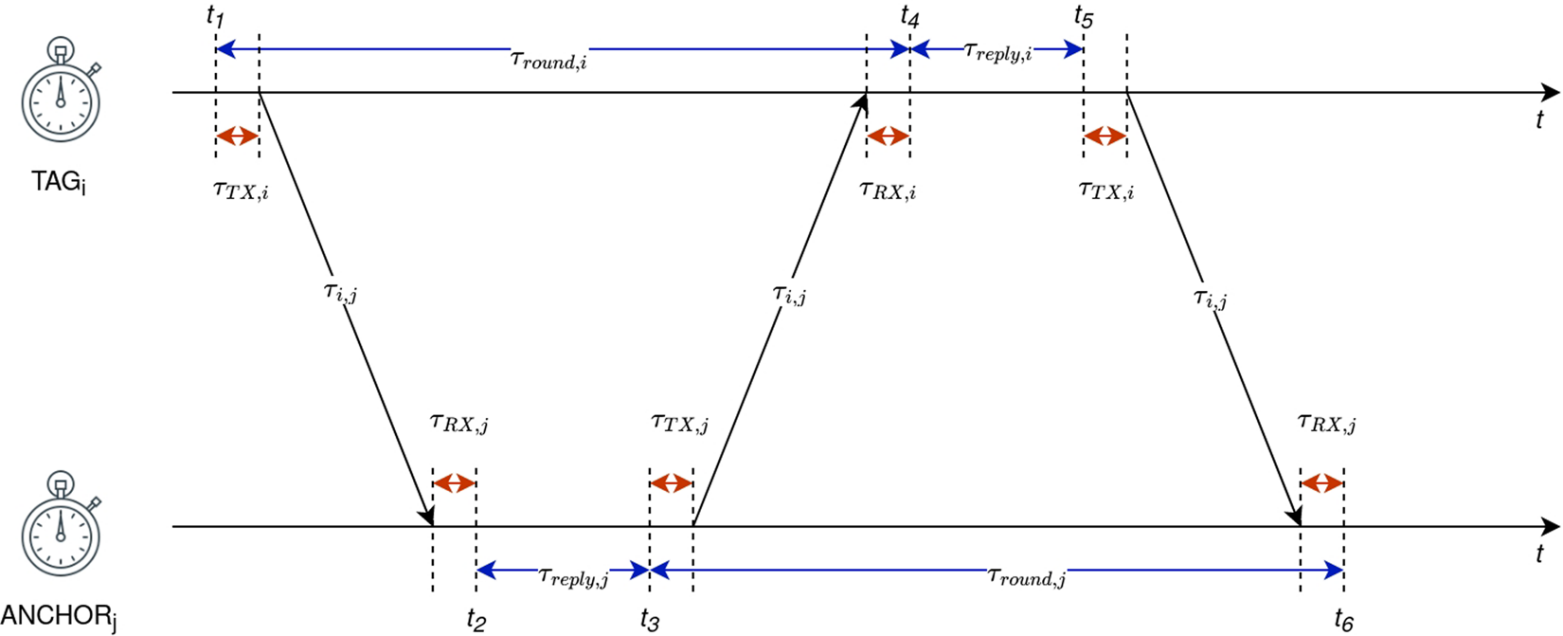
ADS-TWR timing presentation with TX and RX antenna delays.

The Eq. (9) in Section UWB Ranging assumes the ideal measurement transceivers without additional hardware-induced delays such antenna delays. In reality, each transceiver poses its own amount of additional delay added to the signal propagation time that is being introduced by the imperfections and variations in propagation path between the transceiver and antenna (inside the UWB tag or anchor device) which is unique for each individual UWB device. A situation is depicted in the Fig. 4, where *TAG*_*i*_ and *ANCHOR*_*j*_ antenna delays are represented as ${\tau }_{TX,i}$ ${\tau }_{RX,i}$, ${\tau }_{TX,j}$ and ${\tau }_{RX,j}$, respectivelly. The manufacturing imperfections of physical antenna implementations and circuitry imperfections lead to different TX and RX antenna delays. RTTs can be now reformulated by introducing antenna delays as presented by Eqs. (10–12)

10 $${\tau }_{round,i}={t}_{4}-{t}_{1}=2{\tau }_{i,j}+{\tau }_{reply,j}+{\tau }_{AD,i,j}$$

11 $${\tau }_{round,j}={t}_{6}-{t}_{3}=2{\tau }_{i,j}+{\tau }_{reply,i}+{\tau }_{AD,i,j}$$

12 $${\tau }_{AD,i,j}={\tau }_{TX,i}+{\tau }_{RX,i}+{\tau }_{TX,j}+{\tau }_{RX,j}$$

where ${\tau }_{AD,i,j}$ is the total antenna delay introduced by the pair *TAG*_*i*_ and *ANCHOR*_*j*_^20^. The ToF equation by introducing antenna delays can now be reformulates as in Eq. (13).

13 $${\tau }_{i,j}\left({\tau }_{AD,i,j}\right)=\frac{{\tau }_{round,i}{\tau }_{round,j}-{\tau }_{reply,i}{\tau }_{reply,j}-\left({\tau }_{reply,i}+{\tau }_{reply,j}\right){\tau }_{AD,i,j}-{\tau }_{AD,i,j}^{2}}{{\tau }_{reply,i}+{\tau }_{reply,j}+{\tau }_{round,i}+{\tau }_{round,j}+2{\tau }_{AD,i,j}}$$

The total antenna delay *τ*_*AD, i, j*_ for each *TAG*_*i*_-*ANCHOR*_*j*_ device pair does not change for the selected device pair during the measurements and can be compensated during data pre-processing if both measured and actual ranges are known for all measurement points. Instead of calibrating UWB devices prior to the data collection, we selected different approach and collected all data with uncalibrated devices. The measured ranges are compensated for the antenna delays during the post-processing phase. Our offset compensation approach is based on the fact that the shortest LoS distance between the tag and each anchor is the least affected by the environmental effects and thus can be used as the reference range offset estimate. The range offset *r*_*off*_ is calculated by subtracting the mean measured range *r*_*m*_ from the reference Euclidean distance *d*_*ref*_ of a tag from the selected anchor for the shortest LoS path.

14 $${r}_{off}={d}_{ref}-{r}_{m}$$

The estimated range offset *r*_*off*_ is then subtracted from all measured ranges for the selected combination [environment, anchor, channel].

#### Removing anchor A6 from the environment2

**Fig. 5:**
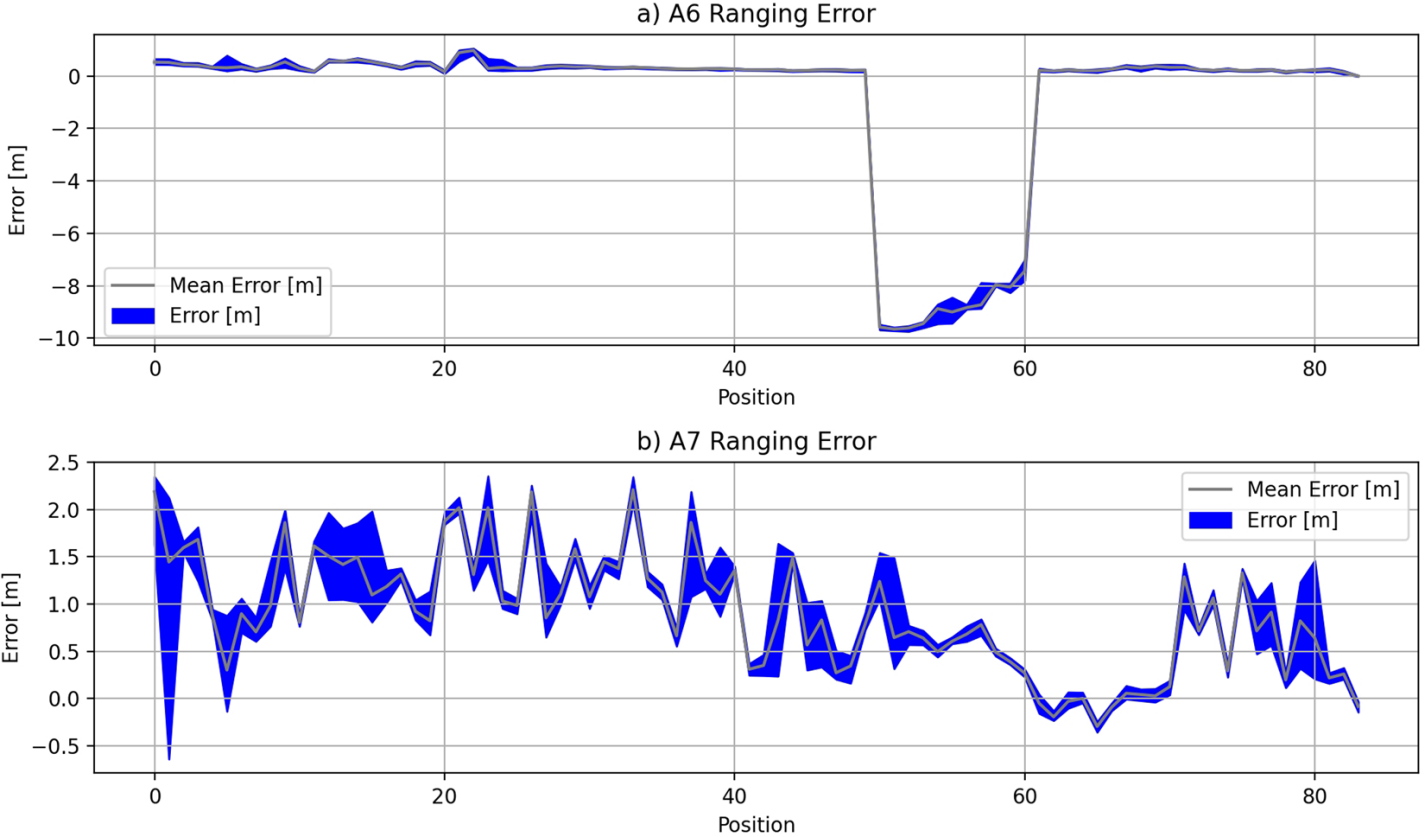
Erratic range measurements for anchor A6 in *environment2* (**a**) compared to expected ranging performance for anchor A7 in *environment2* (**b**). Ranging error for anchor A6 change abruptly from the range of well under 1 meter to the range of 10 meters.

During the data analisys stage, the erroneous measurements were identified in data from the *environment2*. Range measurements were showing erroneous values for measurements taken between position 50 and position 61 for measurements taken with anchor A6 in the environment *environment2*. The total count of positions with erroneous range measurements with anchor A6 in *environment2* is 11. The issue with A6 data in *environment2* is depicted in Fig. 5 where comparison between A6 and A7 is depicted.

The reason for the erroneous range measurements for anchor A6 in *environment2* is the accidental displacement of anchor A6 during the data collection procedure. The anchor A6 fell off the mounting point on the wall inside the industrial environment and the UWB signal propagation path changed in a way the equipment was giving measurements that were far off the expected Euclidean distance between the tag’s position and anchor A6. The measurements are not acceptable to be included in the data set because the measurements with anchor A6 inside the *environment2* were not done in a consistent manner for all the positions on the walking path.

To remove the effects of erratic range measurements on the positioning performance, we completely removed the measurements for anchor A6 in *environment2* from the data set. By removing all A6 measurements from *environment2*, the number of samples collected in a data set for the *environment2* was reduced by 15624. Also, the number of available anchors inside the *environment2* was reduced by one. Data from other three environment were not affected by this issue.

## Data Records

The data set is deposited at Zenodo^21^. Data records described in this paper are deposited in a repository entitled UWB Positioning and Tracking Data Set^21^ in a form of compressed archive including both raw data in *.csv* files and harmonized and pre-processed data in a form of *.json* files that can be easily imported in a programming environment such as Python.

**Fig. 6:**
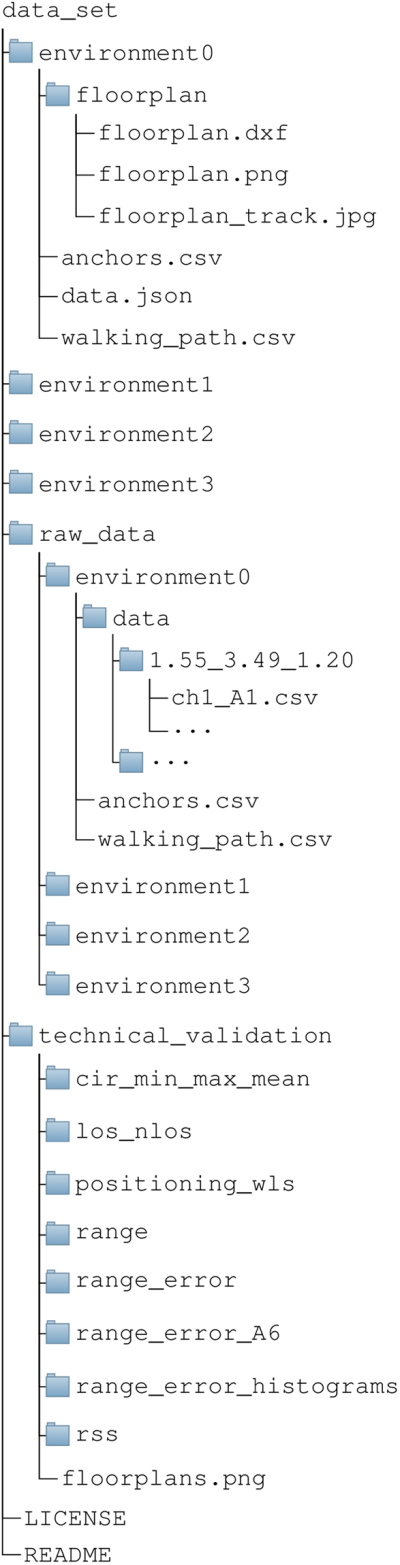
Data set folder structure.

The archive is named *data_set.zip*. The main directory in the archive *data_set* contains complete data set content, whose hierarchical structure is presented in Fig. 6. The *data_set* directory contains five folders: *environment0*, *environment1*, *environment2*, *environment3*, *raw_data* and *technical_validation*. The first four folders contain all measurement-related data for each indoor environment. The fifth folder *raw_data* contains all *.csv* files with raw measurements and the last folder, *technical_validation* contains results from technical validation scripts that are published separatelly in a GitHub repository^27^. Python code for related technical validation, data loading and data analysis together with produced results and trained models for ranging error estimation are part of GitHub repository^27^. The folder content is complemented with a *README* file and *LICENSE* file. Data set is licensed under the Creative Commons Attribution 4.0 International Public License^28^.

Each from the four environment folders contains an *anchors.csv* file with a list of positioning anchors (*A1-A8*) with their corresponding positions inside the selected environment. Each anchor position is presented by *x-axis*, *y-axis* and *z-axis* position in meters. Next file is *walking_path.csv* which contains simulated walking path recorded as a list of tag device positions with values *x-axis*, *y-axis* and *z-axis* in meters. The coordinate system of tag positions is the same as coordinate system of anchor positions. Simulated walking path visualizes the positions of tag device contained in the measurement campaign. Next item in the environment-specific folder is *.json* file *data.json* which contains all measurements collected in a particular environment. The environment-specific folder contains also a subfolder *floorplan* that contains floorplan presentations for the particular indoor environment.

First file in the floorplan presentation subfolder is *floorplan.dxf* file which is an AutoCAD Drawing Exchange Format (DXF) interchangeable file format with a drawing of the indoor environment that can be opened with AutoCAD or any other drawing software supporting DXF format (e.g. LibreCAD etc.). Selected indoor environment floorplan is also represented with file *floorplan.png* which is an image in a Portable Network Graphics (PNG) file format that represents the content of *floorplan.dxf* file. The *floorplan.png* image enables the interested researchers to view the floorplan of selected environment without installing additional software that supports the *DXF* file formats. The folder contains also an image with a simulated walking path overlayed over the floorplan and is named *floorplan_track.jpg*.

The *raw_data* folder contains all measurements collected in a particular indoor environment. For each tag position noted in file *walking_path.csv*, there is individual subfolder with all measurements for the particular tag position. Folder names are formatted as *x-pos_y-pos_z-pos* where the individual axes are of fixed format, e.g. *1.55_3.49_1.20*. Each tag position folder contains 48 measurement files, each file containing measurement for a particular channel and achor pair. There are 8 anchor devices and 6 channel options as noted in Table 2. The file name is defined with a format *chn_Am.csv*, where *n* represents channel number and *m* represents anchor number. File with measurements between tag and anchor device A1 on channel CH1 is therefore defined as *ch1_A1.csv* and, for example, file with measurements between tag and anchor device A6 on channel CH5 is defined as *ch5_A6.csv*.

**Fig. 7:**
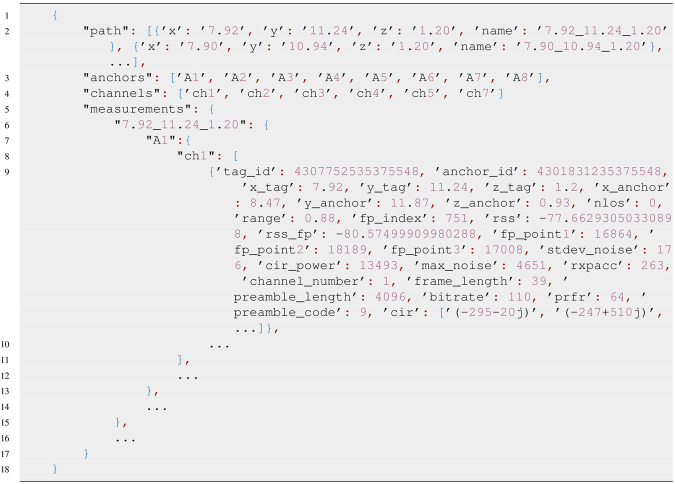
Listing of .json file data structure.

The *.json* file contains organized and parsed data that can be easily accessed and traversed inside all software tools and programming languages that support interfacing data in *.json* format. The organization of data in *.json* file is presented in Fig. 7. First item in *.json* file represents tag positions on a simulated walking path with key name *“path”*. Path item contains a list of dictionary items with individual tag position presentations with keys “x”, “y” and “z” representing x, y, and z axes of individual tag positions and a key “name” that contains a string with tag position name that can be used to access corresponding measurements data inside the *.json* file. Next item in *.json* file is “anchors” that contains a list of anchor names that can be used to access corresponding data under the position name inside the *.json* file. It is followed by the “channels” item that contains a list of channel names that can be used to access the data inside the *.json* file. The last item in *.json* file is “measurements” key, that contains actual measurements organized in hierarchical way starting with position name key, followed by anchor name key and channel name key.

By referencing the data with “position_name”->“anchor_name”-> “channel_name” a list of measurements for selected tag position, anchor and channel is accessed. Each measurement is a dictionary with many “key-value” items. The members of each measurement are listed in Table 3.

**Table 3:** Member keys in a measurement item.

Member	Description
tag_id	Tag ID
anchor_id	Anchor ID
x_tag	Tag position on x-axis
y_tag	Tag position on y-axis
z_tag	Tag position on z-axis
x_anchor	Anchor position on x-axis
y_anchor	Anchor position on y-position
z_anchor	Anchor position on z-position
nlos	nLoS situation: True if nLoS otherwise False
range	Measured range
fp_index	Index of sample at the detected start-of-frame position inside the CIR
rss	Measured RSS value for the received UWB packet (max value)
rss_fp	Measured RSS value for the first path detected in the CIR
fp_point1	Absolute value of first CIR point from the fp_index position
fp_point2	Absolute value of second CIR point from the fp_index position
fp_point3	Absolute value of third CIR point from the fp_index position
stdev_noise	Standard deviation of noise measured at the UWB packet reception
cir_power	Max absolute value of CIR
max_noise	Max value for noise detected at the UWB packet reception
rxpacc	Number of preamble symbols accumulated during the CIR estimation procedure
channel_number	Selected channel number
frame_length	Frame length
preamble_length	Preamble length setup
bitrate	Selected bitrate
prfr	Pulse repetition frequency
preamble_code	Preamble code selected for the communication
cir	List of CIR values in a complex form

Folder *technical_validation* contains results of technical validation of data set. Subfolder *cir_min_max_mean* contains the figures from all indoor environments, where CIRs for each anchor and channel are visualised in a form of mean CIR with max CIR and min CIR envelope. The following subfolder *los_nlos* contains figures representing LoS CIR and nLoS CIR for anchor *A1* on channel *ch1* in *environment0*. Subfolder contains results from analysis of positioning performance based on WLS multilateration with ranging error estimation which is beyond this manuscript and is based on theory from past work^29^. The context is introduced in the section *Usage Notes*.

Subfolder *range* contains figures presenting comparison of actual ranges and measured ranges for each individual anchor and channel inside the four indoor environments. Subfolder *range_error* contains similar figures as in *range* but presenting the ranging errors instead of raw ranges. It is followed by the subfolder *range_error_A6* which specifically present the erroneous situation with anchor *A6* in *environment2*. It is followed by a subfolder *range_error_hisograms* which contain ranging error histograms for individual indoor environments and a comparison between *environment2* and *environment3* as is presented in the manuscript. The last subfoder in *technical_validation* is subfolder *rss* which contains figures with RSS values in relation to range for individual anchors and communication channels. Last item in the *technical_validation* is image *floorplans.png* with all four indoor environments.

## Technical Validation

We assessed the quality of data and its usability for the purpose of positioning algorithm development.

### Channel impulse response analysis

**Fig. 8:**
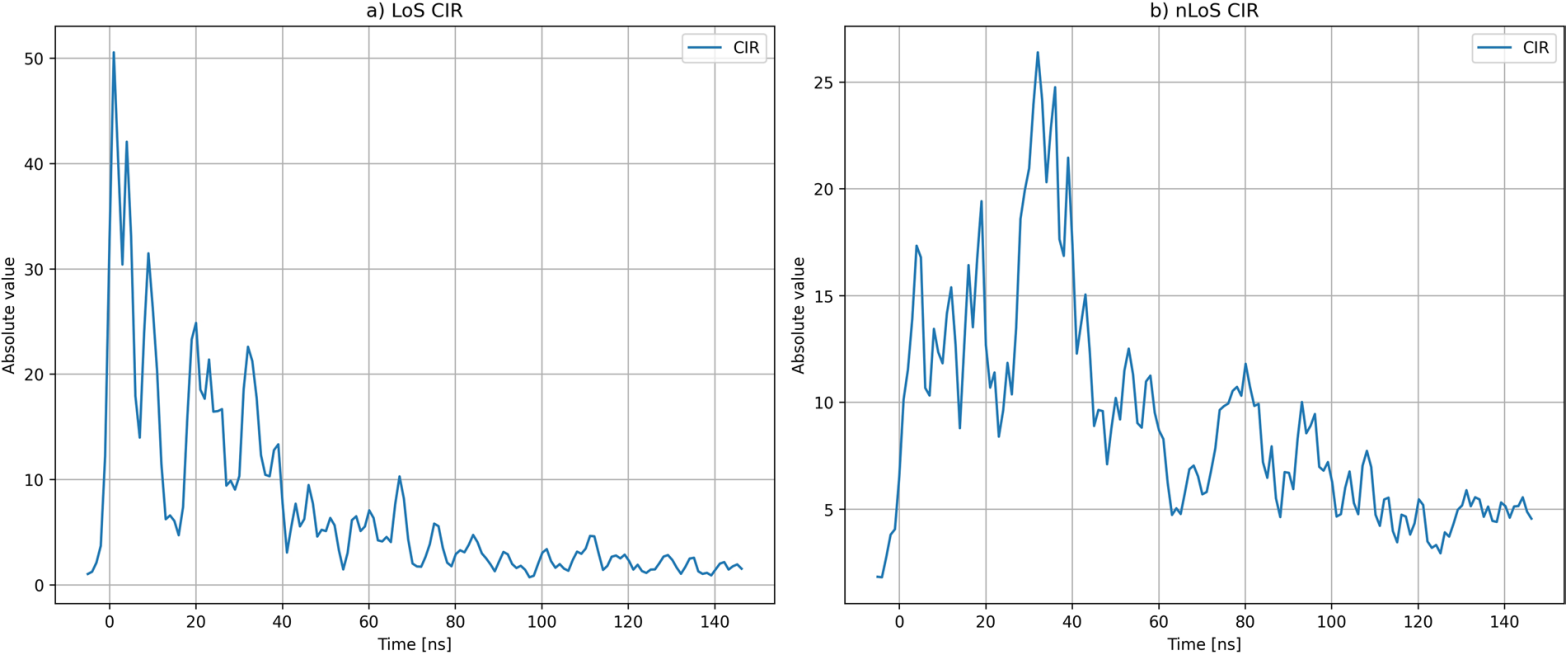
CIR for LoS channel condition (**a**) and CIR for nLoS channel condition (**b**).

The high temporal resolution of UWB technology enables UWB equipment to distinguish between the direct path signals and multipath components during the reception. CIR is a piece of information that is being provided by the DW1000 UWB transceiver for each received UWB message and can be used for the assessment of the channel quality for the particular range measurement. The quality of particular range measurement depends on factors such as signal-to-noise ratio (SNR) and LoS. In case of LoS between the UWB devices, the first received path of the signal represents the direct propagation path of UWB signal, but in the case of NLoS, the first path of a received signal is a multipath signal that travelled longer distance by the reflections. A comparison of LoS signal to the NLoS signal is presented in Fig. 8, where the part a) represents the LoS situation and the part b) represents the NLoS situation. Both CIR examples are from the *environment2* with anchor *A1* on channel *ch1*, where LoS CIR example represent mean CIR at position 8 and nLoS CIR example represent mean CIR at position 61. The differences between LoS and nLoS CIRs can be the source of information for development of algorithms for detecting LoS and nLoS channel conditions^29^.

### RSS analysis

The data set includes RSS value for all measurements in the field named *rss*. The DW1000 UWB transceiver does not contain a dedicated RSS measurement unit but enables the estimation of RSS by using the Eq. (15), where frame quality assessment values from the DW1000 registers derived from the CIR are used for the RSS estimation^24^.

15 $$RSS[dBm]=10\times {\log }_{10}\frac{C\times {2}^{17}}{{N}^{2}}-A$$

*C* in Eq. (15) is the CIR power value reported in the DW1000 registers provided by the leading edge detection algorithm, *A* is the constant used for the RSS estimation which os 113.77 for the PRF of 16 MHz or 121.74 for a PRF of 64 MHz and *N* is the RX preample accumulation count (RXPACC) value holding the number of received preamble symbols for the internal CIR estimation also provided as a value in DW1000 registers and in data set in field *rxpacc*^24^.

According to the DW1000 documentation^24^, RSS estimation based on Eq. (15) is very close to the actual RSS when receive level is low (below −85 dBn) but shows lover levels for higher receive levels which makes the RSS characteristic non-linear. The effect of the RSS non-linearity is even greater in case of the data set because the selected transmit power levels vere selected in a way to cover greatest possible distances between the devices which pushes great part of LoS measurements deep into the non-linear region.

**Fig. 9:**
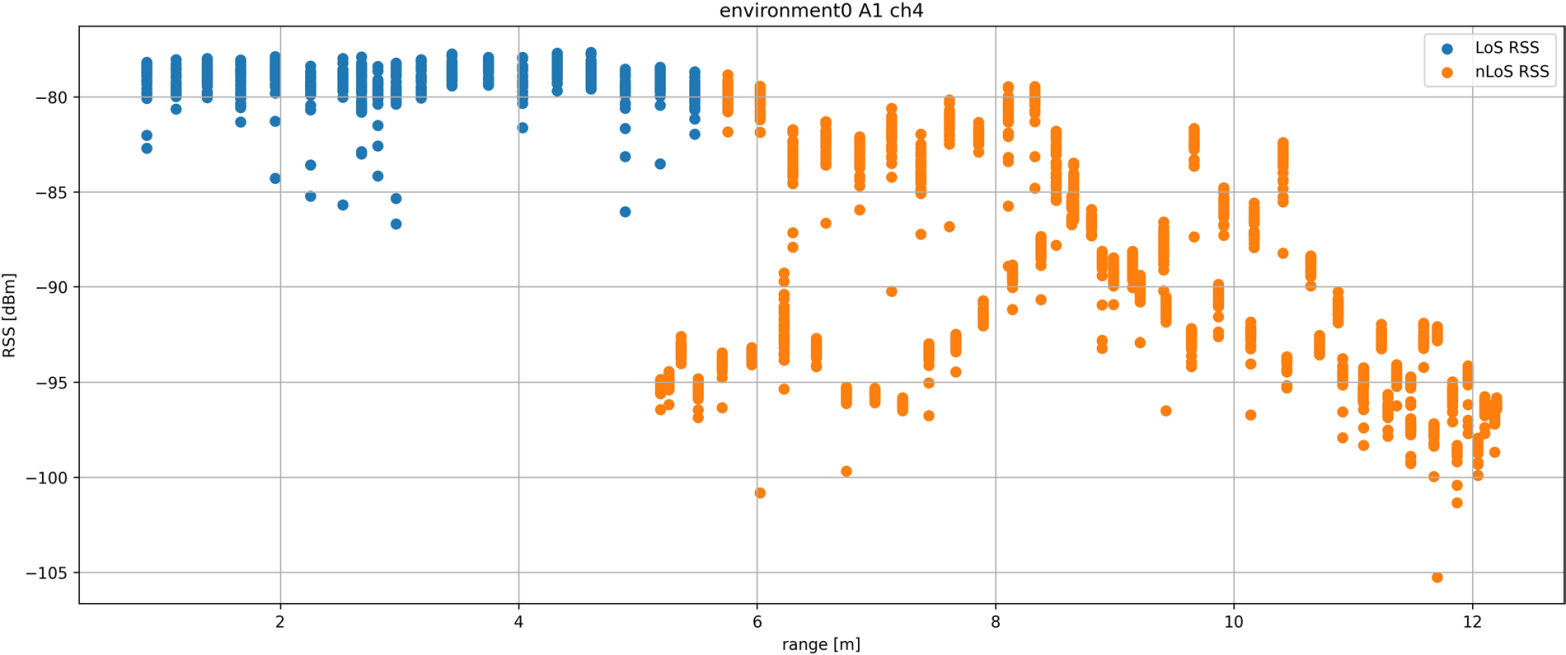
Graph of RSS measurements in *environment0* with anchor *A1* on channel *ch4* for LoS and nLoS positions in relation to ranges.

That makes the data set unsuitable for development of high-precision positioning systems based on range estimation from the RSS. Figure 9 presents a situation from the *environment0* for anchor *A1* on channel *ch4*. RSS values for LoS measurements under the range of 5 m stop increasing because of the RSS estimation method saturation which make them unsuitable for range estimation for shorter ranges. NLoS RSS measurements on the other hand also do not resemble linear function between range and RSS values. In nLoS conditions, the RSS values are less reliable than UWB ranging information, because RSS values change more with the severity of nLoS condition due to the higher attenuation with more severe signal obstructions. In the case presented in Fig. 9, tag passes the same ranges from the anchor twice: first with two plaster walls as a signal obstruction (greater signal attenuation), and second, with one plaster wall as a signal obstruction (weaker signal attenuation).

Situations for all environments, anchors and communication channels are collected in the data set folder inside the *technical_validation* subfolder.

### Ranging error analysis

Ranging error increases with the severity of LoS obstructions and with the increasing distance between the devices. Visual obstructions from the materials that are transmissive for RF signal does not block the UWB signals completely but just change their propagation velocity and attenuate them. The obstructions that are more severe and thus less transmissive to the UWB signals prevent UWB signals to continue on the predefined path and block their propagation. Blocked UWB signals can be deflected to other propagation paths which are longer than the starting propagation path. Part of the UWB signal also reaches the destination by diffraction. All those effects increase the propagation delays of original UWB signals and disperses the propagation delays of multipath components in time which as a result increase the measured range by ToF measurements in nLoS conditions. We call the difference between the measured range and the actual range a ranging error.

**Fig. 10:**
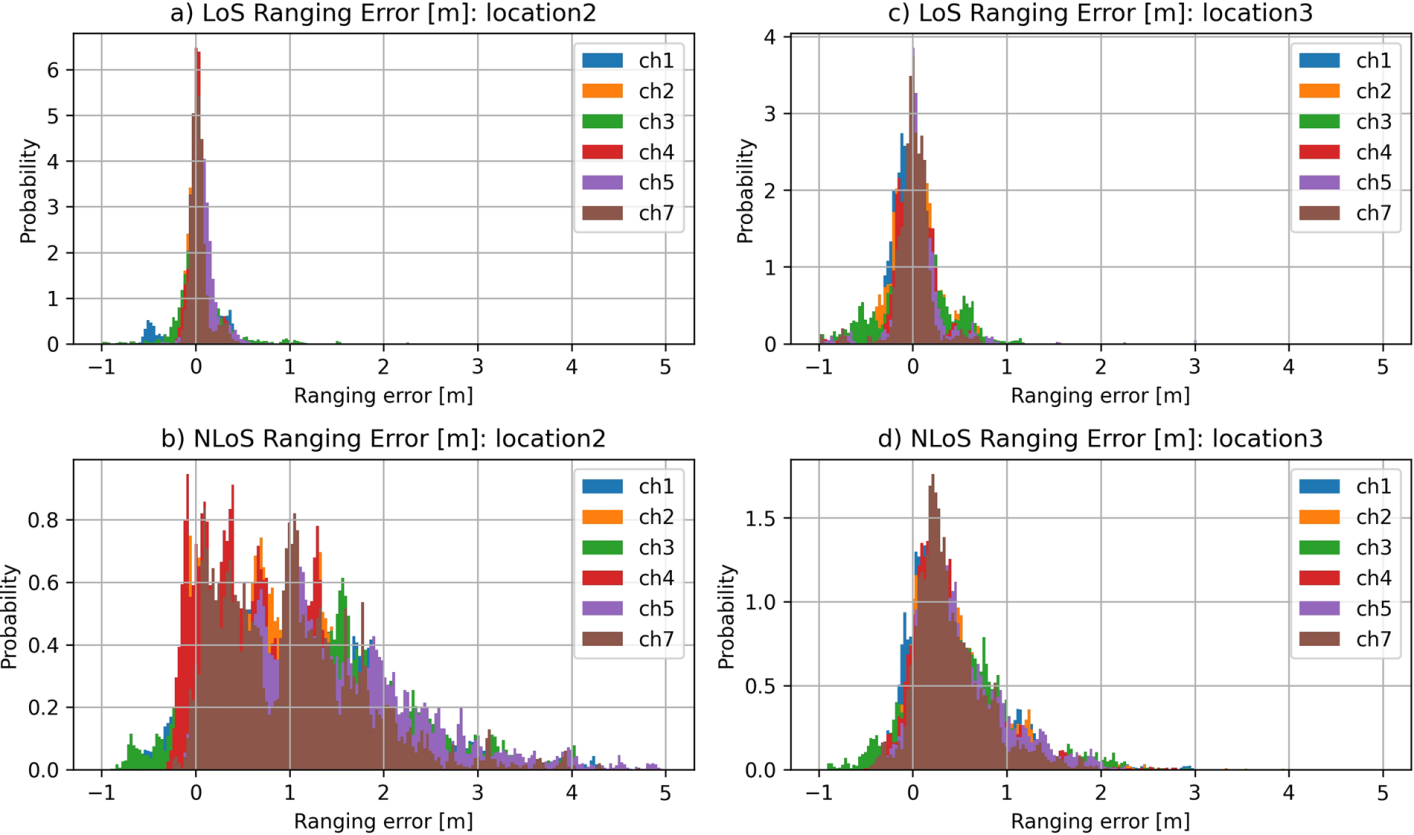
Ranging error histograms for (**a**) LoS conditions in *environment2* (industrial environment), (**b**) nLoS conditions in *environment2*, (**c**) LoS conditions in *environment3* (office environment) and (**d**) nLoS conditions in *environment3* separated into histograms for individual channels.

In the environment with greater distances and more reflective and blocking obstacles, the expected dispersion of ranging errors in nLoS conditions is greater. To demonstrate the effect of environment on the ranging error dispersion, ranging error histograms were created for all four indoor environments. In Fig. 10 ranging error histograms for industrial environment (*environment2*) and office environment (*environment3*) are presented. The ranging error histograms are split into two histograms per environment. First histogram presents ranging errors for LoS conditions and the second histogram presents ranging errors for nLoS conditions. The dispersion of ranging errors in nLoS conditions is higher in industrial environment (Fig. 10b) compared to the office environment (Fig. 10d). The difference comes from the fact that industrial environment contains many metallic and therefore highly reflective surfaces that impact the propagation of UWB signals. Many nLoS signals are being reflected to other reflective surfaces which extends the actual propagation time (distance) of the signal. Office environment contrary to the industrial environment contains very few metallic parts and surfaces and is made of plaster walls. Office environment contains higher portion of materials that are transmissive for the UWB signals. Therefore, nLoS propagation in office environment is less affected by the reflections and more by the attenuation caused by the propagation through the transmissive materials. That introduces smaller ranging errors but also reduces the available range of communication in nLoS conditions because attenuation created by transmission through the walls and furniture is higher than attenuation created by the signal reflections.

## Usage Notes

Data set is primarily created to be used for the development of range-based UWB positioning systems with possible tracking capabilities by using for example Kalman filter. Four walking paths recorded in four indoor environments are included in the data set. Motion-induced artefacts are being eliminated by collecting the data for a pre-defined equi-distant positions on a walking path. All measurements for a selected position are recorded in a way as they will be collected instantly. This ensures all physical distances between anchors and a tag device are constant and invariable during the measurement procedure for one position.

The data can be used for positioning with multilateration approach and techniques for improving positioning accuracy by using different techniques for nLoS detection and ranging error estimation^29^. To improve the positioning accuracy for the moving subject extended Kalman fiter (EKF) can be used^30^. Various approaches and strategies can be developed and evaluated on data set, where improvements to raw range measurements can be made based on the various techniques for selecting the most suitable combination of anchor nodes for individual position estimation. Improvements can also be made by introducing machine learning (ML) algorithms for detecting specific conditions in UWB channel state or for ranging error estimation.

A data set folder in addition to four subfolders with data includes also a subfolder named *technical_validation* where results from technical validation procedures are collected. The technical validation software is published separatelly as a GitHub repository^27^ where all Python scripts are collected with Docker files and instructions for reproduction of results. Those scripts implement several data set quality evaluations. For all four indoor environments a visualization of CIRs is made, all measured ranges per anchor-tag pair are made against the true (reference) ranges, ranging error is being visualized directly and by ranging error histograms and received signal strength (RSS) is being visualized against the true distances. In addition to data set analysis operations positioning by using weighted least squares (WLS) is also included.

Positioning by WLS is based on ranging error estimation using convolutional neural network on CIR information. A script for training the ranging error estimation models is a part of software repository. A ML framework for training the neural networks for the example presented is a TensorFlow framework. Convolutional neural network (CNN) in the example is small enough to be trained on a normal laptop without a dedicated graphics processing unit (GPU). After training the ranging error models, a WLS positioning demonstration can be used that graphically present a comparison of positioning performance between a normal least squares (LS) positioning approach without ranging error mitigation and WLS approach where estimated ranging errors are used as weights in the positioning process.

None of the previosly mentioned procedures like training and calculations are needed to explore the results because all models and resulting graphs are already included in the folder *technical_validation*. The interested reader can explore individual techniques at his own will if he wants to dig deeper but can also explore the results without running all the experiments that are time and resource consuming. The provided techniques can help interested researchers to have a kick-start into the field with all the necessary components without recreating the experiments by themselves.

The reproducibility of the results is guaranteed with the complete processing stack provided in a Docker container that is tested on *Ubuntu 22.04 LTS* and both supports machines with GPU as well as those without GPU available. The user has to install the Docker environment and build the Docker image using the tool *docker compose*. The user is also advised to have a machine that has 32 GB of RAM available to be able to run all the experiments without any interrupts. All instructions for the usage of the provided Docker image setup are provided in the main *README* file inside the data set folder.

## Data Availability

The code that was used to preprocess the raw recorded data such as procedures to fix tag positions, remove range outliers, range offset compensation, removing measurements with A6 in *environment2* and creating cleaned-up data set is published as a separate GitHub repository^27^ and archived with Zenodo to provide permanent access to a usable instance of code. All code is available under the terms of Apache-2.0 License.
